# Control of Advanced Cancer: The Road to Chronicity

**DOI:** 10.3390/ijerph8030683

**Published:** 2011-03-01

**Authors:** Agustin Lage, Tania Crombet

**Affiliations:** Centre of Molecular Immunology, P.O. Box 16040, Havana City 11600, Cuba; E-Mail: taniac@cim.sld.cu

**Keywords:** advanced cancer, chronic treatment, small molecules, biotechnology

## Abstract

Despite the recent trend toward a slight decrease in age-adjusted cancer mortality in some countries, crude mortality rates will continue to increase, driven by the demographic shift towards an aged population. Small molecules (small molecules and biologics) are not only a new therapeutic acquisition, but the tools of a more fundamental transition: the transformation of cancer from a rapidly fatal disease into a chronic condition. Antibodies and cancer vaccines can be used for a long time, even beyond progressive disease, and in aged patients, usually unfit for more aggressive conventional treatments. However, this transition to chronicity will require novel developmental guidelines adequate to this kind of drugs, for which optimal dose is not usually the maximal tolerated dose, pharmacokinetics does not define treatment schedule, and tumor shrinkage is not a good correlate of survival. The ongoing cancer immunotherapy program (including several monoclonal antibodies and therapeutic vaccines) at the Centre of Molecular Immunology can illustrate the issues to be addressed, both biological and social, along the path to transform advanced cancer into a chronic non-communicable disease compatible with years of quality life.

## 1. Introduction: The Increasing Burden of Advanced Cancer

For decades, scientific community has been obsessed in finding a cure for cancer. Modern therapeutic technologies have in fact produced cures, especially in early stage tumors; and the fraction of the incidence which can be diagnosed in early stage has been increased by improved diagnostic technologies.

These technological advances, together with the simultaneous decrease in cigarette smoking, have started to translate, in the last 20 years, into a slight decline in age-adjusted mortality rates. Although the epidemiological landscape varies among different countries, the basic trends are best observed where detailed statistics in hundred million size populations are available. In the US, death rates from all cancers peaked in 1990 for men and 1991 for women, showing then a decreasing trend of 1.3%/year for men and 0.5%/year in women [1]. In Europe, the mortality rate is declining in some countries, but increasing in others [2].

This trend toward decreased cancer mortality is emerging just in some industrialized nations. Among the 7.9 millions of cancer related deaths occurring worldwide in 2007, 70% occurred in developing countries, where mortality continues to increase, cancer is diagnosed late, and the rate of cancer survival is very low.

Nevertheless, the fact that the age-adjusted cancer mortality rate for all tumors, and particularly for some of them is decreasing in some countries, shows that this goal is, at least technically reachable.

Although these achievements have received strong publicity and elicited justified optimism, it should not be forgotten that the age-adjusted mortality rate was what declined and not the crude mortality rate. The slight decrease of the cancer mortality rate within each group of ages is counterbalanced by the demographic trend to an increase in the percentage of population in older age groups. Worldwide the number of cancer deaths per year continues to increase, having doubled in the past 30 years, and it is expected to rise to 17 million by 2030 [3]. Every year, more people die from cancer than from AIDS, tuberculosis and malaria all combined.

The increase in human life expectancy has a second, less noticed, effect on cancer mortality, for the group of patients already diagnosed and apparently cured. This effect comes from the fact that the transition from the disease-free interval (after primary treatment) to the actively progressing disease has probabilistic first grade kinetics [4]. With the delay of mortality from all other causes combined, many successfully treated patients will live more time, at a constant probability rate to relapse.

In addition to the bigger incidence of advanced cancer, the trend towards an increased survival after the disease became advanced, will cause an increase in the prevalence (incidence x survival) of patients bearing advanced cancer.

The overall conclusion is that, no matter what we could advance in primary prevention and early diagnosis, we should expect an increasing burden of advanced cancer in the foreseeable future to be assisted by Public Health Systems. To face this challenge, the significant statistics are prevalence and crude mortality rate, not age-adjusted rate.

Another dimension of the challenge is the prevalence of co-morbidities. The median age of cancer diagnosis is increasing and it has been projected that 70% of all neoplasms will occur in individuals of 65 years and older by 2030 [5]. Within this age group, there is more than 40% probability to find hypertension and heart-related diseases [6]. As many as 25% of 65–69 years old and 50% of 80–84 years old, are affected by two or more chronic health conditions simultaneously [6]. Many of these patients will not be eligible for the current aggressive treatments now considered “state-of-the-art” in Oncology [7].

## 2. The Emerging Evidence of a Transition to Chronicity

Overall figures of incidence and mortality usually blurry the particular epidemiology of the “advanced disease”. The improvement in the long term survival rate of cancer patients reflects the combined effects of earlier diagnose, better therapeutic technology for the loco-regional disease and finally, better outcome of the advanced disease. Trends in mortality rates mix also the effect of changes in primary incidence due to prevalence variation of risk factors such as tobacco smoking.

There are scarce population-based data on survival and mortality once the disease became systemic. Nevertheless, although there is a therapeutic stagnation in some tumors, for several others, survival is increasing inside this stage of the clinical course.

Recent data from the US National Cancer Institute [1] indicates that for already diagnosed cancers with distant dissemination, 5-year survival is 27% for female breast, 28% for oral cavity & pharynx, 31% for ovary, 32% for prostate. Analogous data in Cuba shows 5-year survivals of 30% for female breast, 32% for uterine corpus, 24% for oral cavity and 20% for colon [8–10]. A similar picture is emerging for recurrent cancer. A study of five successive cohorts of patients recurring in 1974, 1980, 1985, 1990 and 1995 shows a continuous trend to a better survival [11,12].

These trends in advanced cancer survival do not contain the effects of improvements on primary prevention, early diagnosis or disease treatment. They should be interpreted as improvements in the systemic treatment of the disseminated disease. Moreover, better overall survival of patients with leukemia, lymphoma, testicular cancer and stage II–III breast cancer, is not attributable to earlier diagnosis but to therapeutic technologies (including prognostic stratification techniques).

With modern therapies, many patients are more likely to die with cancer than of it. The transition from a rapid fatal disease to a chronic condition, compatible with many years of quality life, is not new in the history of Medicine. It is exactly what happened with diabetes mellitus after the discovery of insulin in 1921. Formerly described as a form of death, rather than as a chronic disease, insulin treatment allowed the survival extension by the middle of the XX century. A series of technological improvements (slow insulin, oral drugs, portable glucose meters, insulin sensitizers) ended in 1993 with the report of the “Diabetes Control and Complication Trial” which demonstrated that intensive therapy delays the onset and progression of long term complications in individuals with type 1 diabetes [13].

Analogous histories could be described for cardiovascular diseases, kidney failure, chronic respiratory diseases and other conditions, which as well as advanced cancer, cannot be cured, but can be controlled for many years. The main target of intelligent human intervention in chronic non-communicable diseases is not cure, but control, which means slow progression, and prevention or delay of complications.

## 3. Building the Tools: The Role of Biologics and Small Molecules

Which *could* be the tools of that intervention? Cytotoxic chemotherapy was a major advance in cancer treatment. Introduced by the middle of XX-century through first generation alkylating agents and anti-metabolites, it has been improved along six decades with an increasing arsenal of new antitumor drugs, drug combinations, and support treatments. Chemotherapy made a major contribution to the cure rate of hematological malignancies, testicular cancer and a few other tumors, and contributed to increased survival in many others. But, for survival effect to translate into mortality rate reduction, the improvement should be large enough to allow the intervention of competing causes of death, however, such long term effect would require long term treatments, which cannot be implemented with cytotoxic chemotherapy.

With the exception of anti-hormone treatments for breast and prostate cancers, the therapeutic arsenal of medical oncology has lacked, until very recently, low toxicity drugs suitable for long term use.

Two major acquisitions are changing this landscape: small molecules and biologics.

Both, small inhibitors and biologics have shown activity in combination with chemotherapy and radiation but also as monotherapy. Cancer’s ‘addiction’ to oncogenes is sometimes so strong that even, brief inactivation of a single oncogene can cause a dramatic and sustained response. The big challenge consists is identifying the population that can be susceptible to each treatment with a simple test.

Although there is a wide overlap between small molecules and biologics, we will discuss them separately, for the sake of clarity.

The first flow of therapeutic improvements comes from the introduction of small molecules targeting specific intracellular receptors, which have had a considerable impact on the management of several neoplastic diseases, such as gastrointestinal stromal tumors, hepatocellular and renal cell carcinomas. For chronic myelogenous leukaemia or gastrointestinal stromal tumours (GIST), imatinib (Gleevec), a tyrosine kinase inhibitor that targets platelet-derived growth factor receptor, KIT, and the BCR-ABL oncoprotein [14,15], is remarkably effective in providing long-term control. Sunitinib (Sutent) and sorafenib (Nexavar) are small inhibitors that target vascular endothelial growth factor receptor, platelet-derived growth factor receptor, C-Kit and others. Antitumor activity has been shown in renal cell carcinoma for sunitinib as first-line treatment and for sorafenib, as second-line. In addition, sunitinib is approved as second-line therapy for patients with GIST refractory to imatinib while sorafenib has resulted in a significant prolongation of survival in patients with hepatocellular carcinoma [16]. Still, two other agents have been approved for the second-line treatment of lung cancer (NSCLC): erlotinib (Tarceva) and gefitinib (Iressa), which can produce a dramatic response in those subjects with activating mutations of the Epidermal Growth Factor Receptor (EGFR) [17,18].

The second flow of therapeutic improvements comes from the so called Biotechnology Revolution. What it means, is that the development of recombinant DNA techniques, modern high scale fermentation and purification technologies, has allowed us to obtain and produce biological molecules with the same purity, scalability and reproducibility of synthetic chemistry pharmaceuticals. The potential diversity of biological molecules is mind-boggling. Looking only at antibodies, a human being can have more than a billion different molecules, and the introduction of point mutations at will, could even increase that figure by several orders of magnitude. Potential pharmaceutical applications are very diverse, but up to now, the main target of Biotechnology has been cancer treatment.

Starting with Interferon alpha, registered in 1995 for malignant melanoma, 16 biologics have so far entered into the market for cancer treatment, including 10 monoclonal antibodies [19], and the first therapeutic vaccine for prostate cancer in 2010 [20].

The last published survey of the Pharmaceutical Manufacturers Association of America shows 633 biologics in development. Out of these, 254 are being developed for cancer treatments, including 109 monoclonal antibodies and 63 vaccines [21]. Assuming that just one third of these drugs, will enter into the market, we could expect more than 80 new biologics for treating cancer in the near future [21].

Currently the monoclonal antibodies which target the CD20 molecule (rituximab), the Epidermal Growth Factor Receptor (cetuximab), the HER-2 receptor (trastuzumab), and the Vascular Endothelial Growth Factor (bevacizumab) are already among the 10 top-selling drugs [22].

The contribution of monoclonal antibodies for the treatment of cancer is not just because they enlarge the arsenal of choices. They are also a different kind of drugs. Their toxicities (although not inexistent) are much lower than those of classic cytostatics, and there is emerging evidence of the benefit of their long term use, even beyond the progression of the disease [23,24].

Antibodies can also be used in elderly or frail patients who are unfit for chemotherapy. These are precisely the properties required for a therapeutic tool to be instrumental in the transition of advanced cancer to a chronic disease. Moreover, there is a huge potential of combining monoclonal antibodies and vaccines with conventional chemotherapy and radiation regimes.

Chemotherapy and immunotherapy were first considered to be non-compatible, because of the immunosuppressive effect of cytotoxic drugs. However, more recent evidence is suggesting that chemotherapy could in fact stimulates antitumor immunity through diverse mechanisms such as the induction of immunogenic apoptosis, the opening of homeostatic space (lymphopenia) for the expansion of antitumor lymphocyte clones, and the inhibition of the regulatory loops of the immune system (regulatory T-cells and myeloid-derived suppressor cells [25–27]. Combinations of biologics with some chemotherapies at low doses, could also be evaluated as therapeutic regimes amenable to chronic use [28,29].

## 4. Using the Tools: The Changing Paradigm in Clinical Research

The old and widely accepted paradigm for oncology drug development was based on the experience of classical cytotoxic agents. Currently accepted concepts on chemotherapy were built in the 70s based on experiments on transplantable tumors in mice and on the early experiences of successful trials mainly in hematological malignancies. These concepts were later extended to solid tumors, without the same success rate. Most cancer pharmacologists would accept that antitumor activity is connected to toxicity and therefore, treatments must be scaled up to maximal tolerated dose (to be found in Phase I clinical trials); that pharmacokinetics is relevant to define the optimal schedule; that an active drug should produce a rapid tumor shrinkage; that response rate is a predictor of survival; and that tumor progression indicates treatment failure. However, the unique characteristics of small molecules and biologics challenge these dogmas and demand novel developmental guidelines. For these new drugs the optimal biologic dose can be far below the maximal tolerated dose, mechanisms of action can be indirect and therefore not directly related to pharmacokinetics, effect in survival can be seen without tumor shrinkage and therapeutic effect could be delayed in time and continue beyond progression. Accordingly, a new paradigm for drug development is emerging after the introduction of these new molecules in the clinic [30–33]. This emerging paradigm aims at finding the optimal biologic dose in a “proof of principle trial” according to some pre-defined biological endpoint or biomarker; followed by an efficacy assessment in a randomized trial with long term treatment and survival as the main endpoint. Efficacy trials could test drug combinations directly and their statistical design should take into account the possibility of delayed separation of survival curves.

The current approach for drug development has other limitations. As per the statistics, as few as 2% to 3% of cancer patients are enrolled in clinical trials [34]. This accrual is not representative of the general population, since trials impose very restricted inclusion criteria. For example, older patients are underrepresented in clinical trials, yet approximately two-thirds of patients diagnosed with non-small cell lung cancer are older than 65 years, and nearly 50% are older than 70 years. In a meta-analysis of patients recruited in clinical trials, only 17% were over 70 years old [35].

As a consequence, the results of the clinical trials might not be reproduced in the real cancer population [36,37]. Randomized clinical trials provide internal validity, yet, whether benefits/safety extend to the general population often remain unknown. Well designed population studies would provide unique insights into the generalization and societal benefits of treatment in the real world. Considering its unique safety profile, it would be feasible to conduct population based studies with small inhibitors or biologics.

## 5. Case Study: The CIM-Immunotherapy Program

There are not many organizations conducting fundamental immunology research, clinical trials and biotechnology manufacturing at the same time. There are not many organizations developing proprietary monoclonal antibodies and therapeutic vaccines at the same time. The Centre of Molecular Immunology (CIM) is one of them. It is an organization with a staff of 1,000 people located in the west Havana Biotechnology Pole in Cuba [38]. The comprehensiveness of the CIM program, its tight links with the National Cancer Program, and the simultaneous priority of the development of Primary Health Care and Biotechnology Industry [38], create a favorable perspective to evaluate how Biotechnology could be instrumental for the transition of advanced cancer into a chronic disease, and the current balance of opportunities and difficulties.

In its 15 years of operation CIM has built a 24 project pipeline including biosimilar molecules such as recombinant Erythropoietin, Granulocyte Colony Stimulating Factor, antiCD20 and anti-HER2 monoclonal antibodies, together with proprietary monoclonal antibodies and cancer vaccines.

Its new product development pipeline, although diverse, concentrates around three main targets: The Epidermal Growth Factor Receptor (EGFR) system, the cell membrane gangliosides and the regulatory loops of the immune system.

Two of the proprietary products have already transited through clinical trials and received registration in several countries: the anti-EGFR humanized monoclonal antibody nimotuzumab [39–41] and the EGF conjugated therapeutic vaccine CimaVax-EGF [42,43]. Two other proprietary monoclonal antibodies, the 14F7 anti-ganglioside antibody [44] for breast cancer and the anti-CD6 antibody (itolizumab) for autoimmune diseases [45], together with two new therapeutic vaccines, the N-glycoylganglioside vaccine for breast cancer [46] and the racotumumab anti-idiotype antibody for lung cancer, are currently in clinical testing [47].

Product development has followed the classic paradigm of pre-clinical pharmacology, phase I–II–III clinical trials and regulatory clearance. However, the clinical experience with these products illustrates three fundamental concepts which are pivotal for the transition of advanced cancer into a chronic disease: chronic use, combinations, and primary care involvement.

The humanized monoclonal antibody nimotuzumab has shown so far antitumor activity (combined with chemo-radiation) in head and neck [40,41,48], brain [49,50] and esophageal cancer (unpublished results). It is currently being tested in 25 clinical trials, ongoing in 12 countries, for other tumor localizations.

It has a very good safety profile, basically devoid of skin toxicity which is a limiting toxicity for other anti-EGFR drugs [51,52]. This property has allowed long term use of nimotuzumab, even in pediatric population. More than 450 patients have received bi-weekly treatment for 1 year or more, and 135 for more than 2 years. Drug related adverse events (grade 1 or 2 toxicity in 15–20% of cases) have shown the same probability of manifestation in the first doses and after 1 year of treatment, indicating the lack of cumulative toxicity.

The CimaVax-EGF therapeutic vaccine has provided survival advantage in advanced non-small cell lung cancer. The survival effect is related to the anti-EGF immune response, and to the decrease of seric EGF concentration [43]. Immunogenicity is improved after using a combined schedule, before and after chemotherapy [53].

Again, long term vaccination is very well tolerated. More than 50 advanced lung cancer patients have received monthly vaccinations for more than 1 year, without evidence of cumulative toxicity. Moreover, continuous vaccination increased the probability of obtaining high anti-EGF antibody titers, a response which is correlated with survival [54].

Both, antibody and vaccine long term use is compatible with administration in the primary care setting. A continuous care program in selected policlinics has been recently launched for the treatment of advanced lung cancer after termination or failure of first line chemotherapy, aiming to integrate immunotherapy, nutritional support and pain control.

## 6. The Obstacles Along the Road

The road to convert advanced cancer into a chronic disease compatible with years of quality life using the tools of biotechnology will face obstacles at three levels. Two of them are operational. The third is rooted in the fundamental biology of the interaction between the immune system and the tumor.

The first operational problem is how to prepare the health system to efficiently manage a growing number of patients with advanced cancer, who will require long duration and complex assistance, but who will be ambulatory and socially integrated. Most modern public health systems emerged at the moment when the control of infectious diseases and maternal and child care were the challenges to face. They are not well prepared for the management of chronic diseases [55,56]. In the control of infectious diseases and the reduction of infant mortality there are a handful of highly protagonic interventions producing major impact at population level (preventive vaccinations, antibiotics, institutional childbirth, maternal lactation, safety of water, availability of rehydration treatments). The management of chronic non-communicable diseases on the other hand, requires complex and long interventions including diverse components, none of which produce high impact in isolation [57]. In the particular case of advanced cancer, anticancer drugs should be integrated with an appropriate palliative care including nutritional support and pain control [58]. About 60% of advanced cancer patients experience several kinds of dysfunction as well as moderate to severe pain requiring opioids. If novel anticancer drugs will slow the progression of the disease and prolong life, then, quality of life becomes a major goal.

A new kind of medical research will be needed, that goes beyond the conventional clinical trial methodology, to evaluate several interventions simultaneously, as a package, for survival and quality of life in unselected patient populations.

The second operational problem is manufacturing and costs. Chronic use of biologics in many patients at primary care level will be impossible with the current costs, which are at odds with the worldwide concern on escalating health expenditures [59,60]. A major component of this problem, which is beyond the scope of this article, is health economics, and the current debate about who pays for what. Another component is manufacturing capacity. Monoclonal antibodies (and also some vaccines) are produced through mammalian cell fermentation, a technology demanding big investments. Just for the currently most used antibodies (targeting CD20, EGFR, HER2 and VEGF) at the existing dosages and schedules, and taking into account the prevalence of their indications, a demand of 2 kg per million inhabitants can be calculated. This translates into a worldwide demand of more than 10,000 kg of monoclonal antibodies, only for cancer treatment (not including other uses like autoimmune diseases). The installed manufacturing capacity in the few countries using this technology is currently less than 3,000 kg. Huge industrial investments or major advances in the efficiency of the process, or both, will be required.

Beyond these operational obstacles, there are also several basic biology problems to be addressed. Three of them worth to be mentioned here: the first is the size and duration of the immune response. The immune system has been selected by evolution for fast but short term response to an invading pathogen. It is endowed with diverse mechanisms of programmed contraction of the response. Therapeutic cancer vaccines will require long lasting immune effectors. Probably, these effectors would be also required by monoclonal antibodies whose antitumor activity is partially dependent on the mobilization of cellular immunity [61]. Chronic immunotherapy will need the combination of monoclonal antibodies and therapeutic vaccines with drugs acting on the redundant regulatory loops of the immune system. Several clinical trials of this kind of combination are emerging in the literature [62,63].

The second is senescence. The *Homo sapiens* species evolved during 150,000 years in a high mortality environment. The current life expectancy beyond 70 years is a feature of the last 200 years of our existence. In fact, human life expectancy has been below 30 years for more than 99% of the time that our species has lived in this planet [64]. This means that natural selection, based on reproductive performance, has not fixed genetic traits assuring health after the reproductive life, precisely the period of high incidence of cancer. Moreover, evolution could have fixed genes which are protective in the early life but deleterious afterward, such as those conditioning systemic inflammation in the senescent immune system [64]. Long lasting immunotherapy for elderly patients with advanced cancer should include therapeutic interventions on the immune-senescence process. Several possibilities are emerging from laboratory animal research [65], but none has moved to the clinical setting yet.

The third is complexity. The immune system is a network of thousands of different molecules and cell types, interacting among themselves. Complexity means that we cannot predict the behavior of the system from the behavior of any of its components studied in isolation. This is a major challenge for the development of therapeutic combinations. Having dozens of antibodies and cancer vaccines (as it will be the case), represent the possibility of hundreds of different combinations and schedules, which are just impossible to test empirically, one by one in clinical trials. Additionally, biological molecules could have dual roles, being immunostimulant in some contexts and immunosuppressive in other, as has been already shown for interleukin-2 and Granulocyte-Macrophage Colony Stimulating Factor [66,67]. A theoretical framework guiding the design of therapeutic combinations will be needed, but it is still not available.

## 7. Conclusions: Moving from “Proof of Concept” to Impact in Public Health

Cancer survival for all stages is improving at a population level. In the US, 5, 10 and 20-year survival rate estimates for all types of cancer were 63%, 57% and 51%, respectively [1]. In Europe, a study of three million cancer patients diagnosed in the period 1995–99, showed a relative 5-year survival rate of 46% for men and 58% for women [68]. In Cuba, the 5-year survival rates for breast cancer (female), colon cancer and prostate cancer have been estimated in 84%, 60% and 69%, respectively [8,9].

Moreover, for some tumors, cancer survival is improving in the recurrent and disseminated stages. In clinical trials, small molecules and biologics have produced survival advantage in several tumors, and the low toxicity of these treatments allows them to be used long term, even beyond disease progression. New and more effective treatments for palliation and symptom control are now available.

These data provide the preliminary evidence that advanced cancer can be transformed into a chronic disease, susceptible to be controlled.

Small molecules and biologics can become new and valuable tools to reinforce the emerging transition to chronicity in advanced cancer. To implement this potentiality, we need to merge the new tools with new concepts. In practical terms, this merging will demand a regulatory framework permissive for direct testing of therapeutic combinations and for evaluation of delayed survival effects; the systematic re-evaluation of new treatments in population-based studies (still rarely sponsored) and in elder and frail patients subgroups (still rarely targeted) more representative of the real cancer patient population.

There is still a long road to be walked between proof of concept and impact in population health. Next steps, as discussed along this review, will depend upon:

- The acceptance of a new clinical trial paradigm (with its regulatory implications) to speed up the registration of new drugs, particularly for cancer immunotherapy.
- The improvement in the manufacturing processes of biologics to guarantee wide availability and lower costs.
- The training of the primary health care institutions to use biologics and small molecules for long term, and to evaluate their impact in the context of complex health interventions, including nutritional and “quality-of-life” interventions.
- The clinical testing of drug combinations, wisely designed on the basis of the diverse mechanisms of action of the new drugs.
- The output of fundamental research, providing tools to intervene in the redundant regulatory circuits of the immune system.
- The identification of a simple set of meaningful tests to personalize the probability of effect of given small molecules or biologics in individual patients.
- The quality evaluation of managing advanced cancer at population level, narrowing the discrepancies between what ought to be done and what is really done.

In advanced cancer, the road to chronicity will still be long, but there is already enough accumulated knowledge to draw a road map.
